# PROTACs: Walking through hematological malignancies

**DOI:** 10.3389/fphar.2023.1086946

**Published:** 2023-02-20

**Authors:** Lara J. Bou Malhab, Habiba Alsafar, Saleh Ibrahim, Mohamed Rahmani

**Affiliations:** ^1^ Research Institute of Medical and Health Sciences, University of Sharjah, Sharjah, United Arab Emirates; ^2^ Center for Biotechnology, Khalifa University, Abu Dhabi, United Arab Emirates; ^3^ Department of Biomedical Engineering, College of Engineering, Khalifa University, Abu Dhabi, United Arab Emirates; ^4^ Department of Physiology and Immunology, College of Medicine and Health Sciences, Khalifa University, Abu Dhabi, United Arab Emirates; ^5^ Department of Molecular Biology and Genetics, College of Medicine and Health Sciences, Khalifa University, Abu Dhabi, United Arab Emirates

**Keywords:** PROTACs, hematologic malignancies, resistance, VHL, CRBN

## Abstract

Proteolysis targeting chimeras (PROTACs) are heterobifunctional small molecules that uses the proteasome ubiquitin system to target proteins of interest and promote their degradation with remarkable selectivity. Importantly, unlike conventional small molecule inhibitors, PROTACs have proven highly effective in targeting undruggable proteins and those bearing mutations. Because of these considerations, PROTACs have increasingly become an emerging technology for the development of novel targeted anticancer therapeutics. Interestingly, many PROTACs have demonstrated a great potency and specificity in degrading several oncogenic drivers. Many of these, following extensive preclinical evaluation, have reached advanced stages of clinical testing in various cancers including hematologic malignancies. In this review, we provide a comprehensive summary of the recent advances in the development of PROTACs as therapeutic strategies in diverse hematological malignancies. A particular attention has been given to clinically relevant PROTACs and those targeting oncogenic mutants that drive resistance to therapies. We also discus limitations, and various considerations to optimize the design for effective PROTACs.

## 1 Introduction

Hematological malignancies are a highly heterogeneous group of blood cancers caused by abnormal differentiation of hematopoietic stem cells. Despite the remarkable advances in targeted therapy in hematological malignancies, chemotherapy is still the most common strategy. However, a major concern of chemotherapy is the side effects and long-term sequelae. Targeted therapies have been primarily employing either monoclonal antibodies or small molecules inhibitors. However, each of these approaches has advantages and disadvantages (Lee et al., 2018; Wilkes, 2018). Monoclonal antibodies are known for their high selectivity, high binding affinity, prolonged pharmacokinetic profile, and efficacy in blocking extracellular protein-protein interactions (Lu et al., 2020). Nevertheless, they are large units, which restrict them from crossing cell membrane and, consequently, their use is largely limited to cell surface targets. In addition, oral bioavailability of monoclonal antibodies is quite limited due to their poor ability to cross the intestinal epithelium and their susceptibility to the proteolytic degradation by digestive enzymes (Singh et al., 2008). On the other hand, small molecule inhibitors can be easily administered orally and are able to target intracellular proteins due to their cellular permeability. However, small molecules inhibitors recognize specific pockets or active sites within the protein targets which are lacking in the majority of human proteins particularly transcription factors, non-enzymatic proteins, and scaffold proteins (Toure and Crews, 2016; An and Fu, 2018).

While a number of new small molecule inhibitors and monoclonal antibodies have shown great activities in various hematological malignancies (Podhorecka et al., 2014; Hou et al., 2021; Sochacka-Cwikla et al., 2021), targeted protein degradation using Proteolysis Targeting Chimeras (PROTACs) has emerged as a promising approach in these as also in other types of malignancies (He et al., 2020a). This strategy exploits the ubiquitin-proteasome system (UPS) to target various proteins of interest for degradation. Notably, several PROTAC compounds have entered clinical evaluations in various tumors including hematological malignancies. While the BTK degrader NX-2127 has recently entered phase 1a/b clinical trial in patients with relapsed and refractory B-cell malignancies (NCT04830137), ARV-471 and ARV-110 have shown promising results in phase1/2 clinical trial in locally advanced or metastatic ER^+^/HER2^−^ breast cancer (NCT04072952) or metastatic castration-resistant prostate cancer (NCT03888612) respectively. In this review, we discuss the recent advances, limitations, and future directions of PROTACs in hematological malignancies.

### 1.1 A glance at the ubiquitin proteasome system

The ubiquitin-proteasome system (UPS) is the main proteolytic system in eukaryotes that controls proteins degradation and regulates different cellular processes such as stress responses, DNA repair, cell proliferation, apoptosis, etc. This has made the UPS a powerful and essential machine in maintaining protein quality control and homeostasis (Park et al., 2020a).

The first step of a protein degradation by UPS is its modification with ubiquitin tag, a signal for recognition and degradation by the proteasome 26S subunit (Park et al., 2020b). The ubiquitination process involves the covalent attachment of ubiquitin to lysine residues on the substrate protein *via* a three steps enzymatic cascade reaction involving the E1, E2, and E3 enzymes. First, ubiquitin is activated by the E1 ubiquitin-activating enzyme (E1) following a covalent linkage between the carboxyl-terminus of ubiquitin and a cysteine residue on the E1 enzyme forming a thioester bond (E1-ubq). Then ubiquitin is transferred to an E2 conjugating enzyme (E2-ubq), and lastly, E3 ligases transfer ubiquitin from the E2 to the substrate. There are three families of structurally and functionally distinct E3 ubiquitin ligases: 1) The Really Interesting New Gene (RING), which constitutes the largest family of E3 ligases. These E3 ligases are multi-subunit complexes that use specific Cullins as central molecular scaffolds to recruit the targeted substrates and bring them in a close proximity to the ubiquitin-charged E2 (E2-ubq) enzymes. In this case, the ubiquitin is directly transferred from E2-ubq to the substrate without the necessity to form a thioester bond with ubiquitin (Mani and Gelmann, 2005). 2) the Homologous to E6-AP Caboxy Terminus (HECT) family: HECT E3 ligases undergo a catalytic cysteine-dependent trans-thiolation reaction with E2-ubq forming an intermediate covalent E3-ubq bond prior to ubiquitin transfer to the substrate (Huibregtse et al., 1995; Scheffner et al., 1995). 3) The RING-Between-RING (RBR) family: These ligases have two canonical RING domains RING1 and RING2 linking an in-between RING (IBR) domain (Wenzel et al., 2011). They need at least four cycles of tagging the substrate to form a polyubiquitin chain allowing its recognition and degradation by the 26S proteasome system (Hershko et al., 1983; Voges et al., 1999; Ciechanover, 2005; Schulman and Harper, 2009).

Protein ubiquitination is a very dynamic and highly reversible process. It is often counteracted by deubiquitinating enzymes (DUBs), which remove the ubiquitin chain from the targeted substrate preventing its degradation (Komander et al., 2009).

### 1.2 PROTACs technology

**FIGURE 1:**
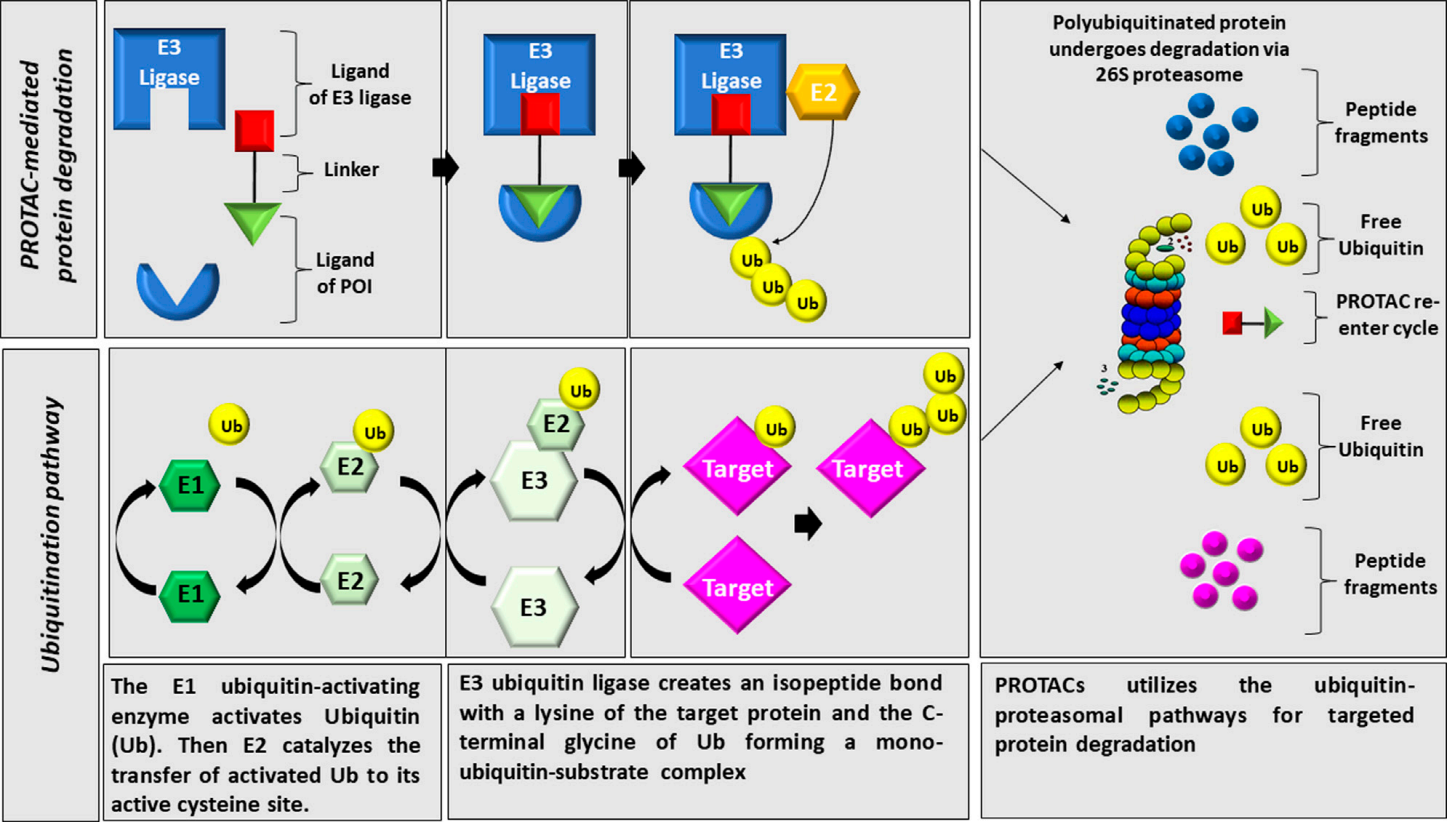
Illustration of PROTAC technology. PROTACs utilizes the ubiquitin-proteasomal pathways for targeted protein degradation through linking an E3 ligase to the protein of interest leading to its polyuiquitination and proteasomal degradation.

Proteolysis targeting chimeras (PROTACs) is a strategy that induces the degradation of target proteins using the ubiquitin-proteasome system as illustrated in Figure 1. PROTACs are heterobifunctional small molecules containing three chemical elements: A ligand that binds the protein of interest, a second ligand that recruits an E3 ubiquitin ligase, and a linker that conjugates these two ligands (Gao et al., 2020). Contrary to small molecule inhibitors, PROTACs technology eliminates the targeted protein through degradation instead of its inhibition (Ciulli and Trainor, 2021). Once the complex (target substrate-PROTAC-E3 ligase) is formed, the E3 ligase employs an E2 ubiquitin-conjugating enzyme to transfer ubiquitin to the substrate. The poly-ubiquitinated substrate will be recognized and degraded by the proteasome system (Paiva and Crews, 2019).

Despite the abundance of E3 ligases (more than 600) expressed in human cells, only few of them have been used in PROTAC technology to degrade target proteins (Fisher and Phillips, 2018). The field has tremendously evolved since the use of the first PROTAC by Sakamoto et al., in 2001 where a poorly permeable phospho-peptide moiety was employed to hijack Skp1-Cullin-F box complex (SCF^ß-TRCP^) to degrade methionine aminopeptidase-2 (MetAp-2) (Sakamoto et al., 2001). In 2003, the same group have developed PROTACs that can target the estrogen receptor-alpha (ER-α) or androgen receptor (AR). These first developed PROTACs were peptide-based with a high molecular weight and very limited cell permeability (Sakamoto et al., 2003). From that point onwards, a number of substrate receptors of E3 ligases were discovered including inhibitors of apoptosis proteins (IAPs), Cereblon (CRBN), and Von Hippel-Lindau (VHL) among others. Many of these have been explored to generate permeable and biologically active PROTACs capable of degrading selected proteins in target cells. The CRBN and VHL have been the most successfully utilized E3 ligase substrate receptors in PROTAC system in hematological malignancies (Ito et al., 2010; Itoh et al., 2010; Buckley et al., 2012a).

#### 1.2.1 Cereblon (CRBN)

**FIGURE 2:**
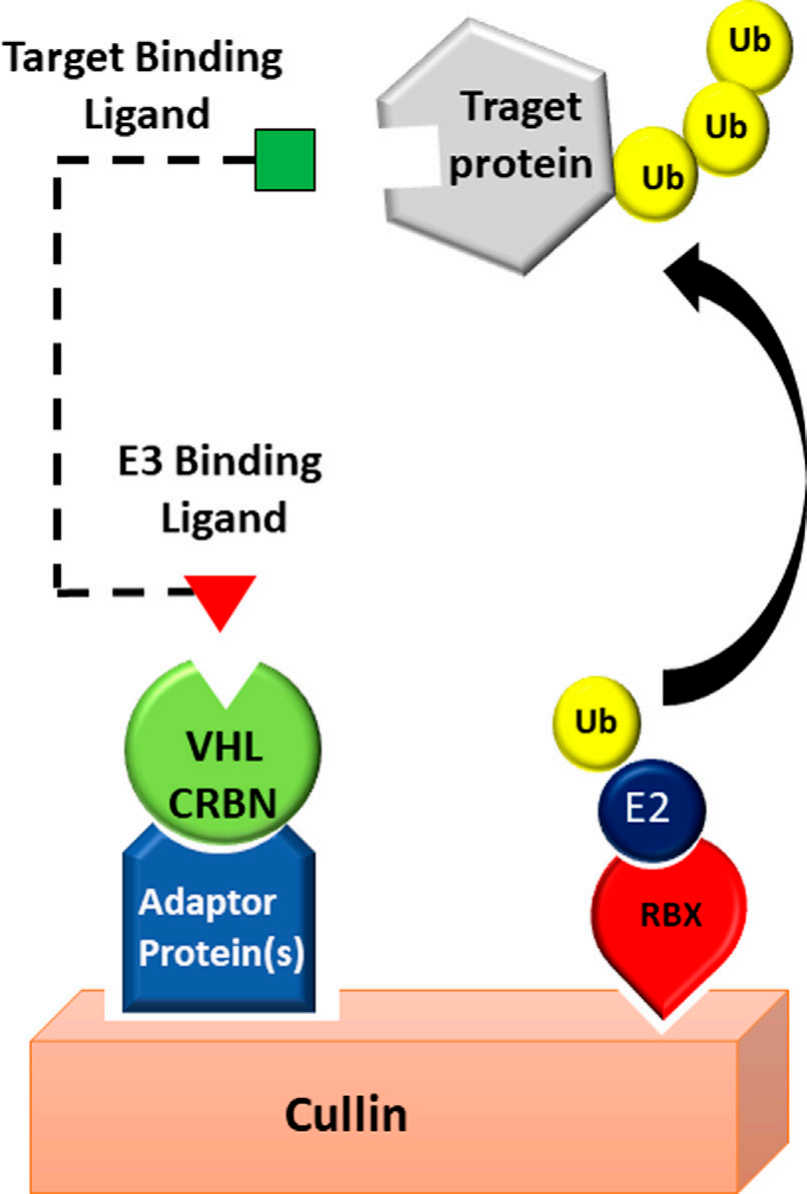
PROTAC technology exploits CRL complex. POI degradation *via* a CRBN or VHL based E3 ligase, where the POI is brought in close proximity to an E3 ligase.

CRBN has successfully been used in the development of PROTAC compounds targeting many proteins in different diseases including various cancers (Bricelj et al., 2021). It is a 442-amino acid protein that acts as a substrate receptor within the Cullin-4-RING E3 ubiquitin ligase (CRL4) complex (Ito et al., 2010). In addition to the molecular scaffold CUL4, this complex also includes the adaptor protein DDB1 (damages DNA-binding protein 1), and the ROC1 protein which recruits the ubiquitin-loaded E2 enzymes (Higa and Zhang, 2007; Jackson and Xiong, 2009; Ito and Handa, 2020) (Figure 2). It is important to note that major advances in this field have been catalyzed by the discoveries from the work of Benjamin Ebert’s group that the direct molecular target of thalidomide and other derived immunomodulatory drugs (IMiDs) such as lenalidomide and pomalidomide is CRBN E3 ligase (Kronke et al., 2014a; Kronke et al., 2014b; Kronke et al., 2015; Sievers et al., 2018). These studies revealed, for the first time, that binding of IMiDs to CRBN leads to increased recruitment of the zinc finger transcription factors Ikaros (IKZF1) and Aiolos (IKZF3) to the E3 complex leading to their subsequent ubiquitination and proteasomal degradation (Kronke et al., 2014b; Chamberlain et al., 2014; Zhu et al., 2014; Kronke et al., 2015). This mechanism is believed to play a major role in the clinical activities of the immunomodulatory drugs (Kronke et al., 2014b; Chamberlain et al., 2014; Kronke et al., 2015).

#### 1.2.2 Von Hippel-Lindau (VHL)

Unlike CRBN which is a part of the Cullin-4-RING E3 ubiquitin ligase, VHL plays a central role in Cullin2 RING E3 ubiquitin ligase complex (CRL2^VHL^), a multiprotein complex containing the molecular scaffold CUL2, elongin B, elongin C, and Rbx-1, also known as ROC1 (Figure 2). VHL is the subunit that binds specifically to the target proteins (Czyzyk-Krzeska and Meller, 2004) and promote their proteasomal degradation. Among many of the substrates targeted by VHL E3 ligase, the hypoxia-inducible factor (HIF)-1α is the best characterized. VHL has been successfully utilized in PROTAC systems to target and degrade many proteins (Sun et al., 2019a). The initially developed VHL-based PROTACs utilized 5 to 7 amino acids long peptides derived from HIF-1α protein (Lee et al., 2007; Schneekloth et al., 2008) as molecular scaffold for the E3 ligase (VHL) instead of small-molecules and they are, therefore, referred to as “bioPROTACs”. The discovery of small-molecule mimetics of the HIF-1α peptide has led to a significant improvement in PROTACs design (Buckley et al., 2012a; Buckley et al., 2012b). One of the first PROTACs using small molecules as VHL-recruiting scaffold was designed to target the bromodomain proteins (BRDs) (Zengerle et al., 2015). In these studies, the bromodomain inhibitor JQ1 was used as BRD4-recruiting scaffold.

IAPs and mouse double minute 2 (MDM2) E3 ligases which are highly expressed in hematological malignancies, have also been of particular interest for the design of PROTACs in this disease and were extensively reviewed elsewhere (Xi et al., 2019; He et al., 2020a).

## 2 Development of PROTACs in hematological malignancies

Over the last two decades, a rapidly growing number of PROTACs have been developed in various hematological malignancies (Table 1).

**TABLE 1:** Representative PROTACs in hematological malignancies.

PROTACs	E3 ligases	Targets	Types of cancer (cells used)	References
DAS-6-2-2-6-VHL	VHL	c-Abl	CML (K562)	(Lai et al., 2016)
DAS-6-2-2-6-CRBN	CRBN	c-Abl Bcr-Abl		
GMB-475	VHL	Bcr-Abl Bcr-Abl G250E	CML (Primary CML CD34^+^, Ba/F3-BCR-ABL1)	Burslem et al. (2019)
SAIS178	VHL	Bcr-Abl Bcr-Abl G250E, V299L, F317L, and F317V mutants	CML (K562)	Zhao et al. (2019)
^PMI^Bcr/Abl-R6	MDM2	Bcr-Abl and various Bcr-Abl mutants	CML; ALL (KU-812, SUP-B15)	Ma et al. (2022a)
P19P	CRBN	Bcr-Abl mutants: V468F, T315I	CML (K562)	Yang et al. (2020)
FLT-3 PROTAC	VHL	FLT-3	AML (MV4-11, MOLM-14)	Burslem et al. (2018)
DT2216	VHL	BCL-xL	ALL (MOLT-4, RS4)	Khan et al. (2019)
PZ15227	CRBN			He et al. (2020b)
753b	VHL	BCL-xL/Bcl-2	AML (Kasumi-1)	Lv et al. (2021)
GT19630 GT19715	CRBN	MYC	AML (HL-60)	Nishida et al. (2022)
PROTAC2	VHL	EED, EZH2 and SUZ12 (PCR2 subunits)	DLBCL (Karpas422)	Hsu et al. (2020)
UNC6852	VHL	EED, EZH2, SUZ12 EZH2-Y641 mutant	DLBCL	Potjewyd et al. (2020)
ARV-825	CRBN	BRD2/3/4	T-ALL, BL (6T-CEM; MOLT-4; Jurkat)	Lu et al. (2015)
MZ1	VHL	BRD2/3/4	AML (Kasumi-1, MV4-11, NB4)	Ma et al. (2022b)
dBET1	CRBN	BRD2/3/4	AML (Kasumi-1, THP-1, MV4-11, NB4)	Zhang et al. (2022)
NX-2127	CRBN	BTK wt BTK C481S mutant IKZF3	MCL, DLBCL	Xie et al. (2021)
NX-5948	CRBN	BTK wild type BTK-C481S mutant	Lymphoma (TMD8)	Robbins et al. (2021)
P131	CRBN	BTK wt BTK C481S mutant	NHL (HBL1, RAMOS, Mino cells)	Sun et al. (2018)
L18I	CRBN	BTK C481S/T/A/G/W	DLBCL (HBL1), MCL (Mino; Z138)	Sun et al. (2018), Sun et al. (2019b), George et al. (2020)
MT-802	CRBN	BTK wt BTK mutants: C481S, E41K, C481R, C481Y, C481T, and C481F.	CLL, BL, DLBCL.	Buhimschi et al. (2018), Lim et al. (2023)
DD-03-171	CRBN	BTK, IKFZ1, and IKFZ3	MCL and other B-cell lymphomas	Dobrovolsky et al. (2019)
MS4077	CRBN	NPM-ALK	Lymphoma (SU-DHL-1)	Zhang et al. (2018)
MS4078		EML4-ALK	NCCLC (NCI-H2228)	
TD-004	VHL	NPM-ALK	Lymphoma (SU-DHL-1)	Kang et al. (2018)
		EML4-ALK	NSCLC (H3122).	

Abbreviations: CML, chronic myeloid leukemias; AML, acute myeloid leukemia; ALL, acute lymphoblastic leukemia; CLL, chronic lymphocytic leukemia; MCL, mantle cell lymphoma; DLBCL, diffuse large B-cell lymphoma; NHL, non-Hodgkin’s lymphoma; BL, Burkitt’s lymphoma; NSCLC, non-small cell lung cancer.

### 2.1 Chronic myeloid leukemia

While several BCR-ABL inhibitors (e.g., imatinib, dasatinib, bosutinib, nilotinib, asciminib) are highly effective in chronic myeloid leukemia (CML) (Wei et al., 2010; Keskin et al., 2016), many patients develop drug resistance due to mutations in the BCR-ABL gene (Hantschel et al., 2012). Efforts aiming at overcoming drug resistance in CML, have led to the development of diverse BCR-ABL PROTACs, some of which have shown great activity and selectivity towards various forms of BCR-ABL including mutants that confer resistance to the BCR-ABL inhibitors. It is important note that these studies have revealed that the capability of a PROTAC to induce the degradation of a given target does not only depend on its capacity to bind to the target, but it is determined by the cooperation of a collection of factors including the inhibitor warhead, the E3 ligase substrate receptor, the nature and the length of the linker, and the point of attachment of the linker on the PROTAC units.

In this regard, Crews group reported in 2016 the first BCR-ABL degraders (DAS-6-2-2-6) based on bosutinib or dasatinib capable of degrading c-ABL and BCR-ABL by using either CRBN or VHL E3 ligase substrate receptors (Lai et al., 2016). These studies revealed that treatment of the CML cell line K562 with 1 uM dasatinib-based DAS-VHL PROTAC led to the degradation of more than 65% of c-ABL protein, but it was ineffective against BCR-ABL. Noteworthy, DAS-VHL effectively engage its target Bcr-ABL as reflected by decreased Bcr-ABL downstream signaling, but did not lead to the degradation of this kinase. Interestingly, when CRBN was used instead of VHL, identical concentration of DAS-CRBN led to the degradation of both c-ABL (>85%) and BCR-ABL proteins (more than 85% and 60% respectively). Few years later, in 2019, new PROTAC called GMB-475 was developed using the GNF5, a small-molecule allosteric inhibitor which binds with high affinity to the myristoyl pocket of ABL kinase. Such compound has the ability to degrade both wild type BCR-ABL and BCR-ABL bearing certain mutations at nanomolar concentrations (Burslem et al., 2019). GMB-475 was particularly effective in degrading BCR-ABL bearing G250E mutation and exhibited a marked antiproliferative activity in cells with such mutation. In addition, GMB-475 exhibited a highly selectivity toxicity toward primary CML CD34^+^ cells, *versus* normal hematopoietic progenitor CD34^+^ cells.

Another BCR-ABL PROTAC, SIAIS178, in which dasatinib was linked to a VHL ligand was developed by Zhao and colleagues (Zhao et al., 2019). In contrast to DAS-VHL PROTAC developed earlier, SIAIS178 showed efficient degradation of BCR-ABL with a DC_50_ value of 8.5 nM and an antiproliferative effect with an IC50 of 24 nM in K562 cells. The differential activity of these dasatinib- and VHL-based PROTACs is probably due to the linker optimization achieved in SIAIS178. *In vivo* studies showed a significant tumor regression following exposure to SIAIS178 in a K562-derived xenograft tumor model. It is important to note that, in addition to its activity in wild type BCR-ABL, SIAIS178 also successfully recognized and degraded several clinically relevant resistance-conferring BCR-ABL mutations such as G250E, V299L, F317L, and F317V, but not T315I. Importantly, Yang et al., reported a series of PROTACs (e.g., P19P) capable of degrading dasatinib-resistant T315I and asciminib-resistant V468F mutations in BCR-ABL (Yang et al., 2020).

In a more recent study, Ma et al. have developed a compound referred to as ^PMI^Bcr/Abl‐R6 that has the potential to degrade BCR-ABL regardless of its mutation status (Ma et al., 2022a). ^PMI^Bcr/Abl-R6 is a dual-targeting PROTAC with a particular design involving an MDM2/p53 inhibitor peptide sequence and Bcr/Abl tetramerization domain. ^PMI^Bcr/Abl-R6 interacts with Bcr/Abl oligomerization domain and binds the ubiquitin E3 ligase MDM2 with high affinity. Consequently, ^PMI^Bcr/Abl-R6 has the potential to degrade all forms of Bcr-Abl (p210, p190, p185) and Bcr/Abl mutants including the T315I, and to activate p53 (Ma et al., 2022a). In fact, ^PMI^Bcr/Abl-R6 showed a significant efficacy in various primary samples isolated from patients with CML and acute lymphoblastic leukemia (ALL), in association with Bcr/Abl degradation and p53 activation (Ma et al., 2022a). Noteworthy, one of these ALL patients bears Y253H, E255K/V, and T315I mutations.

### 2.2 Acute myeloid leukemias (AML) and acute lymphoblastic leukemias (ALL)

#### 2.2.1 FLT-3 PROTACs

FLT-3 gene mutations are the most common mutations in acute myeloid leukemia (AML) (Kiyoi and Naoe, 2002). Midostaurin and gilteritinib are two FDA approved FLT-3 inhibitors especially for patients with FLT-3 mutated AML (Antar et al., 2020). Unfortunately, due to the development of secondary resistance (McMahon et al., 2019), high doses of the inhibitor should be administered to achieve an efficient clinical response which creates off-target toxicities (Grunwald and Levis, 2013). A recent study by Burslem et al., has described a VHL-recruiting FLT-3 PROTAC employing the FLT-3 inhibitor quizartinib. Such compound exhibited high efficiency in degrading FLT-3 ITD in MV4-11 and MOLM-14 AML cells both *in vitro* and in *in vivo* xenograft model (Burslem et al., 2018).

#### 2.2.2 BCL-xL/BCL-2 PROTACs

Apoptosis is a well-characterized and highly regulated mechanism of cell death involving both mitochondrial and non-mitochondrial pathways (Hafezi and Rahmani, 2021). Any deregulation in this mechanism leads to several diseases including tumorigenesis (Plati et al., 2011). Mitochondrial apoptosis is regulated by protein-protein interactions among BCL-2 family members, which control mitochondrial outer membrane permeabilization (MOMP). BCL-2 family members are divided into two functionally and structurally distinct groups: Antiapoptotic proteins (BCL-xL, Mcl-1, Bcl-W, and BFL-1/A1) and pro-apoptotic proteins (BIK, BIM, BID, BAD, BMF, HRK, NOXA, PUMA, BAX and BAK) (Hafezi and Rahmani, 2021). Various BCL-XL or BCL-xL/BCL-2 inhibitors were developed (e.g., Navitoclax also referred to as ABT-263), however, most of them engendered on-target and dose-dependent platelet toxicities as a consequence of the essential role that BCL-xL plays in human platelets survival.

To overcome platelets toxicity of these inhibitors, a PROTAC approach based on VHL or CRBN and ABT-263 have led to the generation of a number of chemically and biologically active compounds among which DT2216 and PZ15227 were the most promising.

DT2216, a VHL-recruiting ABT-263-based PROTAC was recently developed by Khan and colleagues (Khan et al., 2019) and has demonstrated greater affinity to BCL-xL, and yet, showed much lower toxicity to platelets compared to the parent compound ABT-263. Importantly, the anti-tumor activity of DT2216 was considerably more potent than that of ABT-263 in AML cells both *in vitro* and in *in vivo* xenograft mouse model. In addition, DT2216 caused only mild reduction in platelet counts and no sign of reactive thrombocytopenia was observed in mice exposed to this agent (Khan et al., 2019). In a subsequent study, the same group has applied a series of modifications involving different types of linker and varying the attachment points on ABT-263 and E3 ligase ligands. These efforts have led to the development of a dual BCL-2/BCL-XL degrader 753b which exhibits considerable increase in potency compared to the BCL-XL targeting DT2216. These studies also provided evidence that the accessibility of lysines on a target protein is critical in determining the selectivity and potency of a PROTAC for such protein (Lv et al., 2021).

Interestingly, similar results were obtained using another ABT-263-based PROTAC PZ15227 which targets BCL-XL to CRBN E3 ligase for degradation (He et al., 2020b). *In vitro* studies using AML cell lines, revealed that in contrast to ABT-263, PZ15227 exhibits a significant selective toxicity toward malignant cells *versus* platelets. This was also recapitulated in *in vivo* xenograft mouse model. Importantly, PZ15227 resulted in only a moderate thrombocytopenia compared to a similar dose of ABT‐263.

The low toxicity of PROTAC compounds to platelets would likely be explained, at least in part, by the low expression levels of the VHL and CRBN E3 ligases in these cells.

#### 2.2.3 Myc PROTACs

Myc is one of the most frequently dysregulated genes in human cancer including hematological malignancies. It is overexpressed through a variety of mechanisms particularly chromosomal rearrangements. Despite that Myc oncogenic potential has been clearly demonstrated for decades, targeting such transcriptional factor has been challenging. It is important to note that PROTAC approach has not been very successful in directly targeting Myc so far, and the most effective way to interfere with Myc activity is to inhibit its transcriptional regulators such as BET bromodomain protein 4 (BRD4). Importantly, some PROTAC compounds such as the CRBN-based GT19630 and GT19715 have shown potent preliminary activity in AML both *in vitro* and *in vivo* mouse model (Nishida et al., 2022). Specifically, GT19630 and GT19715 effectively degraded c-Myc protein in HL-60 cells with an IC50 1.4 nM and 1.8 nM respectively. In a xenograft mouse model with HL-60 cells, very low dose GT19630 (0.3mg/kg/bid) resulted in a marked c-Myc degradation and tumor growth inhibition. Of note, GT19715 showed greater activity in venetoclax resistant MV4-11 cells, which exhibit increased c-Myc level, compared to venetoclax-sensitive parental cells (Nishida et al., 2022).

#### 2.2.4 BRD PROTACs

The bromodomain and extraterminal (BET) domain protein family which includes BRD2, BRD3, BRD4 has been linked to the development of many tumors including hematological malignancies (Zuber et al., 2011; Alsarraj and Hunter, 2012; Segura et al., 2013; Asangani et al., 2014; Valent and Zuber, 2014; Liao et al., 2016; Wu et al., 2021). These considerations have prompted the search for small molecule inhibitors against BRD4 and some of these compounds have been leveraged to generate PROTACs against this oncogenic factor. Among these, ARV-825 PROTAC in which the BRD4 inhibitor OTX015 was linked to the E3 ligase CRBN binding inhibitor pomalidomide. AV-825 was highly effective in inducing BRD4 proteasomal degradation (Lu et al., 2015). In addition to BRD4, ARV-825 also induces the degradation of BRD3 and BRD2 in T-ALL cells. Importantly, ARV-825 suppressed T cell acute lymphoblastic leukemia (T-ALL) cell proliferation *in vitro via* cell cycle arrest and apoptosis. Such effect is more potent than those of BRD4 inhibitors such as JQ1, dBET1, and OTX015. ARV-825 was also very effective in reducing tumor growth in xenograft mouse model. Mechanistic studies have revealed that ARV-825 inhibited cell proliferation through BET and c-Myc depletion *in vitro* as well as *in vivo* (Wu et al., 2021).

A very recent study in AML reported a highly promising PROTAC compound MZ1, which targets and efficiently degrades BRD2, BRD3, and BRD4 proteins in various AML cell lines, and markedly suppress tumor growth in a xenograft mouse model (Ma et al., 2022b). MZ1 also downregulates cMyc which is positively regulated by BRD (Ma et al., 2022b). Another recently developed CRBN-based PROTAC dBET1 has also shown very potent activity in degrading BRD2, BRD3, and BRD4 proteins in association with a potent anti-tumor activity in various AML cell lines (Zhang et al., 2022).

### 2.3 Lymphoma

#### 2.3.1 PROTACs targeting BTK

BTK plays an essential role in B cell receptor (BCR) mediated B cells activation and proliferation (Mohamed et al., 2009). Ibrutinib, a BTK inhibitor, has been approved for the treatment of mantle cell lymphoma (MCL) and has been clinically evaluated as monotherapy or in combination in several malignancies including activated B cell-like (ABC) DLBCL (Pan et al., 2007). Unfortunately, MCL patients often develop drug resistance to ibrutinib due to C481S missense BTK mutation (Woyach et al., 2014). Recently two CRBN-based PROTAC BTK degraders NX-2127 and NX-5948 have entered clinical evaluations in a phase 1a/b study in patients with relapsed and refractory B-cell malignancies, whose disease progressed after at least 2 prior lines of therapy (NCT04830137 and NCT05131022 respectively).

In 2018, two PROTACs for ibrutinib-resistant BTK degradation were developed, P131 and L18I (Sun et al., 2018; Sun et al., 2019b). These studies, showed that these CRBN-based PROTACs were capable of degrading both the wild-type and ibrutinib-resistant C481S BTK protein at low concentrations. Interestingly unlike ibrutinib, BTK PROTACs showed a potent antiproliferative activities in cell bearing C481S BTK mutant (Sun et al., 2018). The antiproliferative activity of these compounds in DLBCL and MCL with wild-type BTK was also superior than that of ibrutinib (Sun et al., 2018). In addition, L18I, which is a second generation of BTK PROTAC, was not only highly effective against C481S BTK protein, but also potently degrades several other clinically relevant C481 mutations in B-cell tumors with a DC_50_ < 50 nM. More importantly, L18I induced rapid regression of C481S BTK HBL-1-derived xenograft tumors (George et al., 2020).

In another study, Crews’s group developed another highly effective BTK degrader, MT-802 (DC_50_: 14.9 nM) (Buhimschi et al., 2018; Lim et al., 2023). In contrast to ibrutinib, MT-802 was able to reduce the pool of active BTK in primary cells isolated from chronic lymphocytic leukemia (CLL) patients bearing C481S mutation (Buhimschi et al., 2018).

A number of other highly active BTK-specific degrader has been recently developed including DD-04-015, which degrade efficiently BTK only after 4 h exposure (Huang et al., 2018). Such compound was further optimized into DD-03-171 which has the ability to degrade the C481S BTK mutant and showed enhanced anti-proliferative effects on mantle cell lymphoma cells *in vitro* (5.1 nM) and significant efficacy towards patient-derived xenografts *in vivo* (Dobrovolsky et al., 2019).

#### 2.3.2 ALK PROTACs

Anaplastic lymphoma kinase (ALK) is a tyrosine kinase receptor found activated due to different genetic alterations (chromosomal translocations, substitution mutations and gene amplification) in many cancers including anaplastic large-cell lymphomas (ALCL) and diffuse large B-cell lymphomas (Webb et al., 2009). The most common ALK genetic alteration in lymphomas is NPM–ALK fusion protein that results from t(2;5) chromosomal rearrangement. Such aberration leads to ligand-independent constitutive activation of this tyrosine kinase. ALK is also frequently rearranged (EML4–ALK) in some solid tumors particularly non-small-cell lung carcinoma (NSCLC). Various ALK degraders were recently developed including MS4077, MS4078, TD-004. These PROTACs are capable of efficiently degrading NPM-ALK in lymphoma as well as EML4-ALK fusion proteins in NSCLC cells in association with a marked inhibition of cell growth (Kang et al., 2018; Zhang et al., 2018).

#### 2.3.3 PRC2 PROTACs

Polycomb repressive complex 2 (PRC2) has been widely linked to hematologic malignancies (Martin-Perez et al., 2010; Takamatsu-Ichihara and Kitabayashi, 2016; Iwama, 2017). PRC2 protein is composed of EZH2 (enhancer of zeste homolog 2), EED, SUZ12, RBAP46/48 and AEBP2 subunits (Herviou et al., 2016). The EZH2 is the catalytic subunit of PRC2. It catalyzes histone H3 methylation, a process in which both EED and SUZ12 subunits are required (Cao et al., 2002). Importantly, Loss-of function mutations in EZH2 and SUZ12 genes, which encode for central PCR2 components of PRC2 are frequently observed in patients with T-ALL (Ntziachristos et al., 2012; Simon et al., 2012; Zhang et al., 2012). Although effective EZH2 inhibitors exists, preclinical results show drug resistance due to the secondary mutations in both wild type and mutant EZH2 alleles (Gibaja et al., 2016). Two different PROTACs were generated using VHL ligand using different EED inhibitors and different linkers. PROTAC2 developed by Bloecher’s group in 2020, lead to a selective degradation of EED, EZH2 and SUZ12 reducing the proliferation of EZH-dependent tumor cells Karpas422 (Hsu et al., 2020). UNC6852, another EZH2 degrader that was developed by James group has shown similar results against the wild type EZH2, and interestingly also against the Y641N mutant EZH2 (Potjewyd et al., 2020).

## 3 Improving PROTACs potency and specificity

Although cell permeability and target selectivity are major limitations of PROTAC system in therapeutics development, such approach has been highly effective in targeting and degrading many targets in various diseases including cancer. It is important to note that: 1) PROTACs are not equally effective in all tissues. For example, in a study by Zorba et al., a CRBN-based PROTAC compound (compound 10) effectively degrades BTK in mice spleen but not in the lungs despite similar compound delivery to both organs (Zorba et al., 2018). This could reflect tissue-specific differences in the expression of E3 ligases or other components of the ubiquitin system. 2) An important consideration when developing a PROTAC is the degree of expression of a particular E3 ubiquitin ligase, not only in the target cells but also in normal cells. It is conceivable that a high expression of an E3 ubiquitin ligase in aberrant cells makes it a good candidate to use in a PROTAC system. However, if normal cells also express high level of such factor the resulting PROTAC may lead to increased toxicity. 3) Another important consideration came from studies by Lv et al., using computational modelling which revealed that the PROTAC complex has highly selective ubiquitination activity toward lysine residues located on a defined position on the target protein (Lv et al., 2021). As both the selectivity and potency of a PROTAC is determined by the selectivity and potency of ubiquitination machinery, it is highly important to keep these considerations in mind when designing PROTACs.

Another important consideration when it comes to PROTAC design is the choice of the warhead which is critical for the potency and selectivity of PROTACs. This has been highlighted by the work from Lai and colleagues (Lai et al., 2016) in which they showed that dasatinib-based VHL-recruiting PROTACs degrades c-Abl but not Bcr-Abl, in contrast, dasatinib-based CRBN-recruiting PROTACs have the ability to target and degrade both c-Abl and Bcr-Abl. However, when the Bcr-Abl inhibitor bosutinib was used as a warhead instead of dasatinib in VHL-recruiting PROTACs, the activity of such PROTAC was lost against both c-Abl and Bcr-Abl (Lai et al., 2016).

## 4 Conclusion and future perspectives

Given the impressive preclinical activities, the wealth of PROTAC compounds diversity, and the numerous clinical trials testing them, such promising technology is poised to become a new therapeutic approach. However, to date most PROTACs are based on either CRBN or VHL. To enhance our ability to effectively and selectively degrade a wide range of protein targets, it would be critical to explore other E3 ubiquitin ligases and better understand the mechanisms of PROTAC off targets. While the PROTAC approach has particularly been focusing on cancer, such approach should also be explored in other diseases.

Overall, further improvement of the specificity and efficacy of these class of compounds will likely be the key for accelerating the development of this strategy in various types of cancer including hematological malignancies and potentially other diseases.
